# The diagnostic value of grey-scale inversion technique in chest radiography

**DOI:** 10.1007/s11547-022-01453-0

**Published:** 2022-01-18

**Authors:** Roberta Eufrasia Ledda, Mario Silva, Nicole McMichael, Carlotta Sartorio, Cristina Branchi, Gianluca Milanese, Sundeep M. Nayak, Nicola Sverzellati

**Affiliations:** 1 Department of Medicine and Surgery, University of Parma, Scienze Radiologiche, University Hospital of Parma, Padiglione Barbieri, Via Gramsci 14, 43126 Parma, Italy; 2 Department of Radiology Diagnostics, Skåne University Hospital of Malmö, Malmö, Sweden; 3 Department of Radiology, Kaiser Permanente Northern California, San Leandro, CA USA

**Keywords:** Chest radiography, Grey-scale inversion, Diagnostic performance of chest radiography, Interobserver agreement

## Abstract

**Purpose:**

We investigated whether the additional use of grey-scale inversion technique improves the interpretation of eight chest abnormalities, in terms of diagnostic performance and interobserver variability.

**Material and methods:**

A total of 507 patients who underwent a chest computed tomography (CT) examination and a chest radiography (CXR) within 24 h were enrolled. CT was the standard of reference. Images were retrospectively reviewed for the presence of atelectasis, consolidation, interstitial abnormality, nodule, mass, pleural effusion, pneumothorax and rib fractures. Four CXR reading settings, involving 3 readers were organized: only standard; only inverted; standard followed by inverted; and inverted followed by standard. Sensitivity, specificity, positive predictive value (PPV), negative predictive value (NPV) and accuracy, assessed with the area under the curve (AUC), and their 95% confidence interval were calculated for each reader and setting. Interobserver agreement was tested by Cohen’s K test with quadratic weights (*K*_*w*_) and its 95%CI.

**Results:**

CXR sensitivity % for any finding was 35.1 (95% CI: 33 to 37) for setting 1, 35.9 (95% CI: 33 to 37), for setting 2, 32.59 (95% CI: 30 to 34) for setting 3, and 35.56 (95% CI: 33 to 37) for setting 4; specificity % 93.78 (95% CI: 91 to 95), 93.92 (95% CI: 91 to 95), 94.43 (95% CI: 92 to 96), 93.86 (95% CI: 91 to 95); PPV % 56.22 (95% CI: 54.2 to 58.2), 56.49 (95% CI: 54.5 to 58.5), 57.15 (95% CI: 55 to 59), 56.75 (95% CI: 54 to 58); NPV % 85.66 (95% CI: 83 to 87), 85.74 (95% CI: 83 to 87), 85.29 (95% CI: 83 to 87), 85.73 (95% CI: 83 to 87); AUC values 0.64 (95% CI: 0.62 to 0.66), 0.65 (95% CI: 0.63 to 0.67), 0.64 (95% CI: 0.62 to 0.66), 0.65 (95% CI: 0.63 to 0.67); *K*_*w*_ values 0.42 (95% CI: 0.4 to 0.44), 0.40 (95% CI: 0.38 to 0.42), 0.42 (95% CI: 0.4 to 0.44), 0.41 (95% CI: 0.39 to 0.43) for settings 1, 2, 3 and 4, respectively.

**Conclusions:**

No significant advantages were observed in the use of grey-scale inversion technique neither over standard display mode nor in combination at the detection of eight chest abnormalities.

**Supplementary Information:**

The online version contains supplementary material available at 10.1007/s11547-022-01453-0.

## Introduction

Chest radiography (CXR) is generally considered entry level imaging to screen many pulmonary diseases with good performance as a screening uptake evaluation [1, 2]. The interface between the bronchial tree, containing air, and structures with no air gives the radiographic image a natural contrast, used to advantage radiological interpreters (author radiologists) to depict abnormal findings [3]. These intrinsic anatomical features, along with continuous technical advancements in the field of digital radiography, have significantly contributed to make chest radiography one of the most requested radiological investigations [1, 4].

Over the last decades, digital chest radiography has iteratively and incrementally improved, with numerous processing tools being developed to support radiologists in the detection of pathological findings [2, 4, 5]. Most of these tools have been implemented to improve nodule detection, including digital tomosynthesis [6–8], dual energy and temporal subtraction techniques [9–11], computer-aided detection systems [12, 13] and dark-field CXR. More recently, dark-field CXR has been demonstrated to be a valuable complementary tool for the assessment of pulmonary infiltrates, cardiomegaly and hemopericardium [14, 15]. Such techniques are not yet widely available, and their use requires further validation. In comparison, the grey-scale inversion technique is universally available, being a built-in feature on most Picture Archiving and Communication System (PACS) display workstations. Based on the evidence that viewing the inverted image (black on white) improves human contrast perception [16], grey-scale inversion has been proposed as a valid supplementary tool to increase the diagnostic accuracy of radiographic imaging [17–21]. In chest radiography, the diagnostic value of inverted images has been investigated mostly for parenchymal nodules [17, 22–26], pneumothorax [20] and rib fractures [27] detection. The clinical advantages of using this display method, however, are still debated, and no general consensus has been reached.

The purpose of this study is to investigate whether the additional use of grey-scale inversion technique improves the interpretation of the main chest abnormalities, in terms of both diagnostic performance and interobserver variability.

## Material and methods

### Ethics statement

This study was approved by the Institutional Review Board of the University Hospital of Parma (Prot. 51059). Given the retrospective nature of the study, informed consent was waived.

### Study group

The study selection criteria were as follows: chest CT examination and CXR obtained within 24 h of each other, in patients older than 18 years of age admitted to the University Hospital of Parma between October 2017 and October 2019. CTs and CXRs images affected by motion artefacts or other technical limitations (e.g. chest structures only partially included within the CT acquisition volume or the CXR projection) were excluded. Chest CT served as standard of reference (CT technique is reported in *Supplementary material*).

### CXR imaging technique

Posteroanterior (PA) and left-lateral (LL) images were obtained with the patient standing up and in full inspiration with three digital radiography systems (Axiom Aristos FX, Siemens Healthineers; Essenta DR, Philips and DigitalDiagnost, Philips). Acquisition parameters were as follows: 125 kV, 1.6 mAs, antiscatter grid with a 180cm focus–detector distance.

Anteroposterior (AP) images were acquired with the patient either lying down or sitting up with two computed radiography systems (Practix 33 Plus, Philips and Practix 300, Philips). Acquisition parameters were as follows: 95–98 kV, 3.2 mAs, with a 120cm focus–detector distance.

**Fig. 1:**
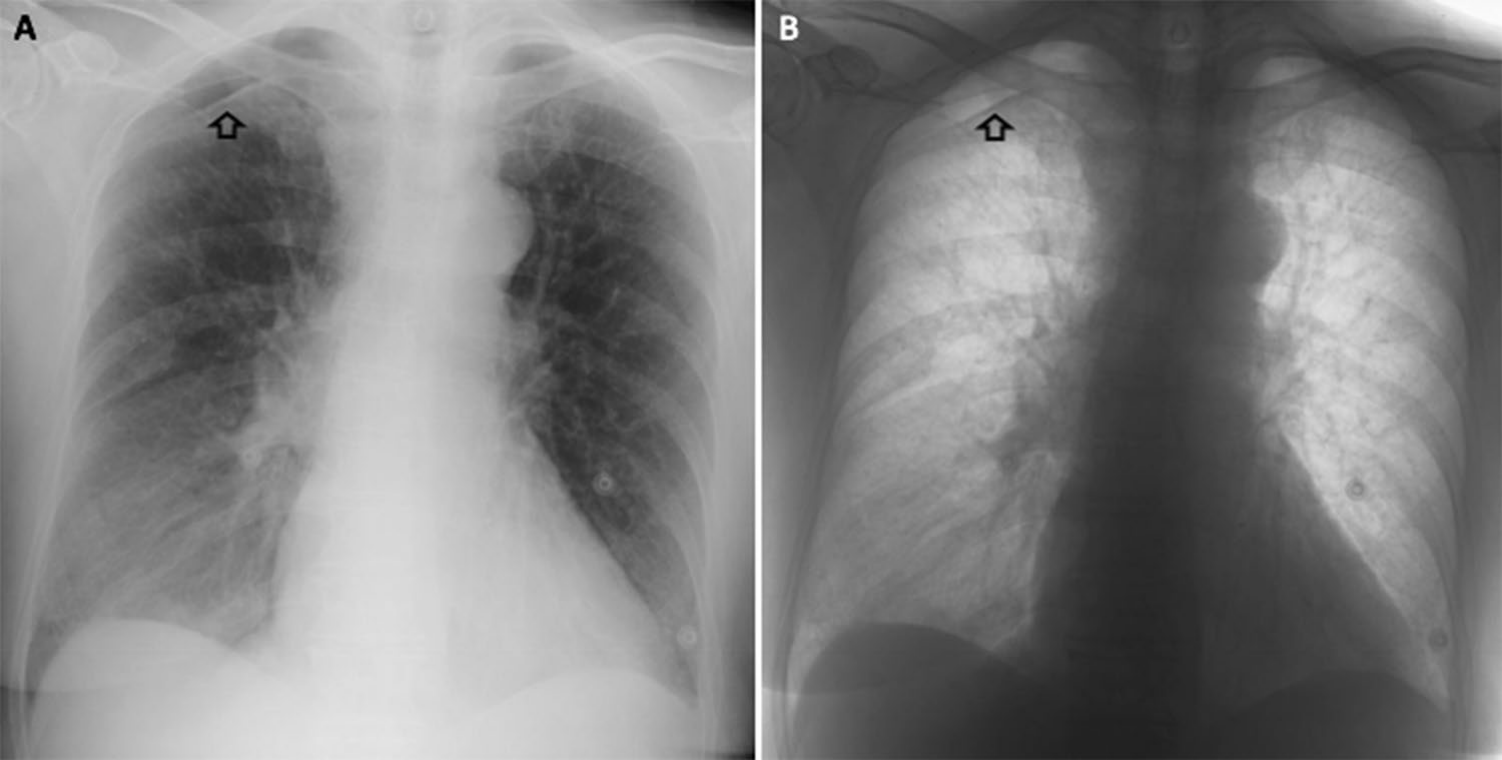
Representative example of right apical pneumothorax (arrows) in standard (**A**) and inverted grey-scale (**B**) CXR images (posteroanterior projection)

**Fig. 2:**
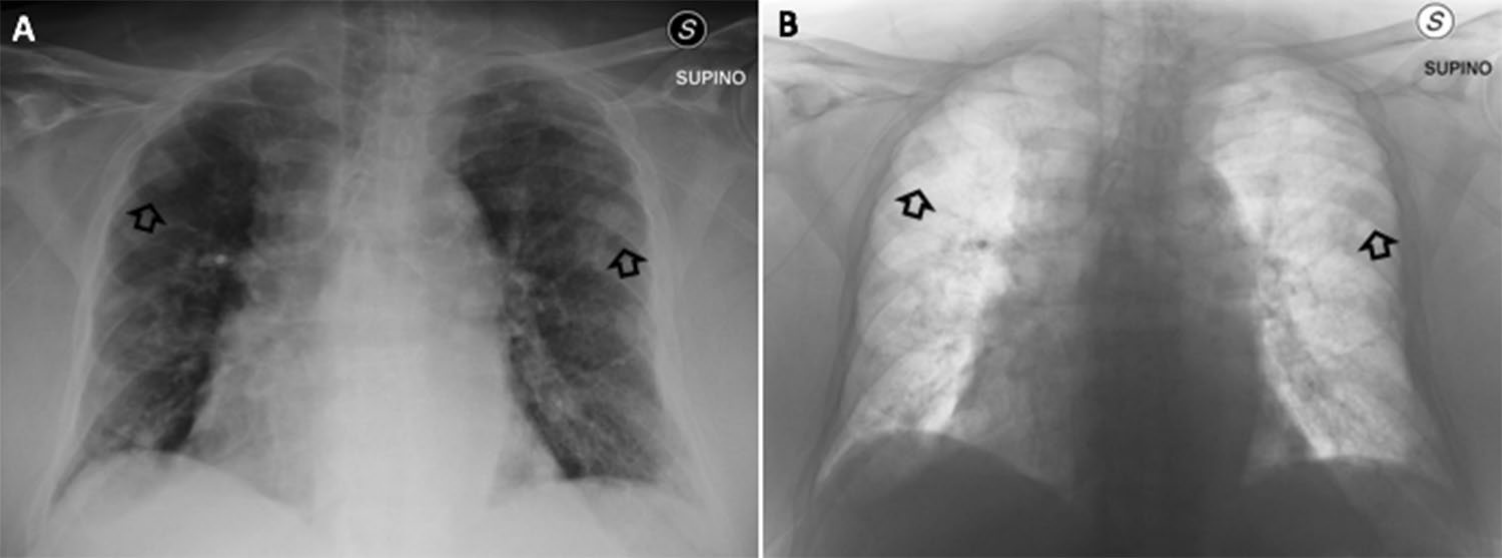
Representative example of bilateral parenchymal nodules (arrows) in standard (**A**) and inverted grey-scale (**B**) CXR images (anteroposterior projection)

Images were visualized on a dedicated workstation (BARCO visualization system, Kortrijk, Belgium), and grey-scale inversion was performed through a built-in software of our PACS workstations (suite Estensa, Esaote, Genova, Italy) (Figs. 1 and 2).

### Data collection and interpretation

*CXR*—Images of CXR were retrieved from the local PACS and independently reviewed by one general radiologist with 18 years of experience (Reader 1) and two third- year radiology residents (Readers 2 and 3), for the presence of eight predefined findings: atelectasis, consolidation, interstitial abnormality, nodule, mass, pleural effusion, pneumothorax and rib fractures. Chest abnormalities were classified based on the Fleischner Society glossary [28]. Standard grey-scale (also called “white bones”) and inverted grey-scale (“black bones”) CXRs were evaluated in two separate reading sessions, as follows:

- Session 1: standard setting first, followed by inverted grey-scale
- Session 2: inverted grey-scale first, followed by standard.

There was a wash out interval of at least 4 weeks between the two reading sessions, and images were evaluated in random order. For each session, annotation of findings was recorded separately for standard and inverted grey-scale to analyse the findings by either first line standard or inverted. Subsequently, the adjunct findings by consecutive reading with either approach were recorded. This database allowed testing of CXR accuracy and interobserver agreement under different reading settings and combinations (see *Statistical analysis*). Reading time was recorded for each reader and session.

*Standard of reference*—The diagnostic performance of CXR with different visualization modes was tested against CT, as standard of reference. CT images were reviewed independently by two resident radiologists (Readers 4 and 5, respectively) who had access to the radiological reports, and classified into positive or negative, as follows:

- Positive CT was assigned in case of at least one of the eight above-mentioned findings;
- Negative CT was assigned when none of them was present.

Any discrepancy between Readers 4 and 5 was resolved by a chest radiologist with 13 years of experience.

The same classification system was applied to discretize CXR outcome in binary categories.

### Statistical analysis

Continuous data were expressed as median and its 95% confidence interval (95% CI), whereas categorical data were expressed as absolute and relative distribution, with corresponding 95% CI using Wilson method.

The following reading settings were assembled for comparison with CT standard of reference:

- Setting 1: standard reading only, derived from session 1
- Setting 2: inverted reading only, derived from session 2
- Setting 3: combined reading, first standard followed by inverted reading as per full session 1
- Setting 4: combined reading, first inverted followed by standard reading as per full session 2

Sensitivity, specificity, positive predictive value (PPV) and negative predictive value (NPV) were calculated for each reader and all reading settings; accuracy was tested with the area under the curve (AUC) values and its 95% confidence interval (95% CI). Interobserver agreement was tested by Cohen’s K test with quadratic weights (*k*_*w*_) and its 95%CI: *k*_*w*_ < 0.20 was considered to indicate poor agreement, 0.21 < *k*_*w*_ < 0.40 fair agreement, 0.41 < *k*_*w*_ < 0.60 moderate agreement, 0.61 < *k*_*w*_ < 0.80 good agreement, and 0.81 < *k*_*w*_ < 1.00 very good agreement.

A *p* value < 0.05 was deemed statistically significant. Statistical analysis was performed by MedCalc Software bvba (version 19.1–64-bit, Ostend, Belgium).

## Results

### Study population

**Fig. 3:**
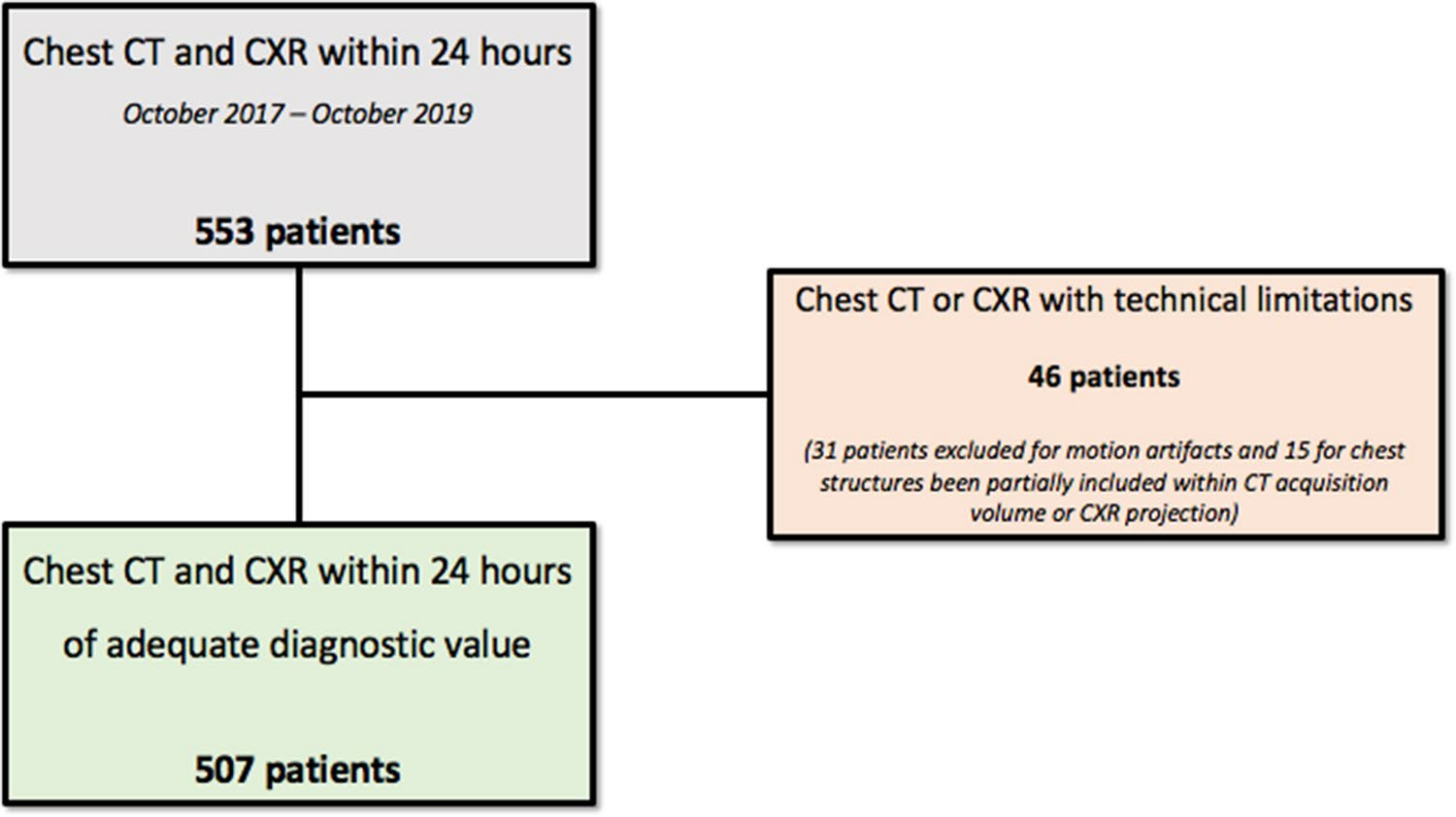
Flow chart of patient selection

A total of 553 consecutive patients underwent a chest CT and CXR within 24 h of each other, at the University Hospital of Parma between October 2017 and October 2019. Forty-six (8.32%, 95%CI 6.3% to 10.92%) patients were excluded because of CT and/or CXR technical limitations: 31/46 (67.39%, 95%CI 52.97% to 79.13%) because of CT motion artifacts; 15/46 (32.61%, 95%CI 20.87% to 47.03%) due to chest structures only partially included within the CT acquisition volume or CXR projections. A total of 507 (median age 69.95%CI 66.94 to 71; 285/507 men, 56.2%) patients were enrolled (Fig. 3). Main clinical indications for both CT and CXR included trauma, chest pain, dyspnea, fever and persistent cough.

### CT findings

A total of 393/507 (77.5%, 95%CI 73.68% to 80.93%) CTs were scored positives and 114/507 (22.5%, 95%CI 19.07% to 26.32%) negatives. Detailed distribution of CT pathological findings is reported in Table 1, whereas CT acquisition data in *Supplementary material*.

**Table 1:** Distribution of chest CT pathological findings

Findings	N°	% [95%CI]
Atelectasis	166/507	32.7 [28.8–36.94]
Consolidation	175/507	34 [30.51–38.76]
Interstitial abnormalities	67/507	13.2 [10.54–16.44]
Nodule (median size 7 mm, 95%CI 6–8)	85/507	16.8 [13.77–20.27]
Mass (median size 55 mm, 95%CI 38.5–73.75)	10/507	1.97 [1.07–3.59]
Pleural effusion	167/507	32.9 [28.99–37.15]
Pneumothorax (median size 23 mm, 95%CI 9.75–35.25)	28/507	5.5 [3.85–7.86]
Rib fractures	65/507	12.8 [10.19–16.01]

### CXR acquisition data

PA and LL projections were performed in 254/507 (50.1%, 95%CI 45.76% to 54.44%), whereas AP projection was performed in 253/507 (49.9%, 95%CI 45.56% to 54.24%). The effect of reading setting was comparable for both standing and supine CXR imaging.

### Reading time

The median reading time of session 1 was 79 s [95%CI, 77 to 85 s] for Reader 1, 84 [95%CI, 80 to 88 s] for Reader 2, and 83 [95%CI, 80.5 to 87 s] for Reader 3, whereas that of session 2 was 61 s [95%CI, 57 to 64 s] for Reader 1 and 59 for Readers 2 [95%CI, 57 to 60.7 s] and 3 [95%CI, 55 to 60 s].

### Diagnostic performance of CXR

Overall, sensitivity of CXR for any finding ranged 7.1–60% for setting 1, and 8.2–60% for each of setting 2, 3, and 4. Specificity ranged 76.5–99.8%, 78–99.8%, 73.3–100%, 73–100%, for settings 1, 2, 3 and 4, respectively. PPV ranged 16.7–90%, 17.2–90.9%, 20–100%, 17.8–100%, for settings 1, 2, 3 and 4, respectively. NPV ranged 69.6–99.2%, 69.3–99.2%, 69.5–99.2%, 69.8–99.2%, for settings 1, 2, 3 and 4, respectively. AUC ranged 0.529–0.781, 0.527–0.779, 0.531–0.779, 0.529–0.779, for settings 1, 2, 3 and 4, respectively. Overall, CXR accuracy was not significantly improved by the inverted images compared to setting 1. For Reader 3, CXR sensitivity was improved by the combined reading at the detection of consolidation in setting 4 and of pneumothorax and rib fractures in setting 3, whereas for Reader 1 the combined approach improved CXR PPV at the detection of pleural effusion in setting 3. Sensitivity, specificity, PPV, NPV and AUC values are detailed for each radiological finding in Table 2.

**Table 2:** Diagnostic performance of CXR—only standard (setting 1); only inverted (setting 2); standard + inverted (setting 3) and inverted + standard (setting 4) (AUC: area under the curve; NPV: negative predictive value; PPV: positive predictive value)

			Atelectasis	Consolidation	Interstitial abnormalities	Nodule	Mass	Pleural effusion	Pneumothorax	Rib fractures
Setting 1	Reader 1	Sensitivity	16.3 [11–22.8]	39.4 [32.1–47.1]	32.8 [21.9–45.4]	9.4 [4.2–17.7]	50 [18.7–81.3]	45.5 [37.8–53.4]	46.4 [27.5–66.1]	29.2 [18.6–41.8]
		Specificity	93.3 [90.1–95.7]	86.8 [82.6–90.2]	95.2 [92.8–97]	97.6 [95.7–98.9]	94.9 [92.7–96.7]	92.4 [89–94.9]	98.5 [97–99.4]	97.3 [95.3–98.6]
		PPV	53.9 [40.9–66.4]	60.5 [52.4–68.1]	51.1 [37.9–64.2]	44.5 [24.6–66.4]	16.7 [8.8–29.3]	74.5 [66.1–81.4]	64.9 [44.5–81]	61.3 [44.6–75.6]
		NPV	69.6 [68.1–71.1]	73.6 [71–75.9]	90.3 [88.7–91.7]	84.2 [83.3–85.1]	98.9 [90.1–99.4]	77.6 [75–79.9]	96.9 [95.7–97.8]	90.4 [88.9–91.6]
		AUC	0.548 [0.50–0.59]	0.631 [0.59–0.67]	0.640 [0.60–0.68]	0.535 [0.49–0.58]	0.725 [0.68–0.76]	0.689 [0.65–0.73]	0.725 [0.68–0.76]	0.633 [0.59–0.68]
	Reader 2	Sensitivity	23.5 [17.3–30.7]	39.4 [32.1–47.1]	35.8 [24.5–48.5]	7.1 [2.6–14.7]	40 [12.2–73.8]	55.1 [47.2–62.8]	32.1 [15.9–52.5]	38.5 [26.7–51.4]
		Specificity	89.7 [86–92.8]	87.7 [83.6–91]	91.4 [88.3–93.8]	98.8 [97.3–99.6]	99.4 [98.3–99.9]	92.7 [89.3–95.2]	99.8 [98.8–100]	96.8 [94.7–98.3]
		PPV	52.7 [42.3–62.8]	62.2 [53.9–69.8]	38.7 [28.9–49.5]	54.6 [27.3–79.4]	57.1 [25.5–83.8]	78.6 [71.1–84.6]	90 [54.1–98.6]	64.1 [49.4–76.5]
		NPV	70.7 [68.8–72.6]	73.8 [71.2–76.1]	90.4 [88.7–91.8]	84 [83.2–84.8]	98.8 [98–99.3]	80.8 [78–83.3]	96.2 [95.1–97]	91.5 [89.8–92.9]
		AUC	0.566 [0.52–0.61]	0.635 [0.59–0.68]	0.636 [0.59–0.68]	0.529 [0.49–0.57]	0.697 [0.66–0.74]	0.739 [0.70–0.78]	0.660 [0.62–0.70]	0.676 [0.63–0.72]
	Reader 3	Sensitivity	48.2 [40.4–56.1]	37.1 [30–44.8]	20.9 [11.9–32.6]	12.9 [6.6–22]	60 [26.2–87.9]	51.5 [43.7–59.3]	42.9 [24.5–62.8]	33.9 [22.6–46.7]
		Specificity	76.5 [71.7–80.9]	91.6 [88–94.3]	95.7 [93.3–97.4]	96.7 [94.5–98.2]	96.2 [94.1–97.7]	88.2 [84.3–91.5]	99.4 [98.2–99.9]	94.1 [91.5–96.1]
		PPV	50 [43.8–56.1]	69.4 [60.3–77.3]	42.4 [27.9–58.3]	44.1 [27–62.6]	24 [13.9–38.2]	68.2 [60.8–74.8]	79.9 [54.4–93]	45.8 [33.8–58.3]
		NPV	75.3 [72.2–78.1]	73.9 [71.5–76.1]	88.8 [87.5–90]	84.6 [83.5–85.7]	99.2 [98.3–99.6]	78.8 [76–81.3]	96.8 [95.6–97.6]	90.7 [89.1–92]
		AUC	0.624 [0.58–0.67]	0.644 [0.60–0.69]	0.583 [0.54–0.63]	0.548 [0.50–0.59]	0.781 [0.74–0.82]	0.699 [0.66–0.74]	0.711 [0.67–0.75]	0.640 [0.60–0.68]
Setting 2	Reader 1	Sensitivity	15.1 [10–2.42]	33.1 [26.2–40.7]	31.3 [20.6–43.8]	8.2 [3.4–16.2]	50 [18.7–81.3]	44.3 [36.6–52.2]	39.3 [21.5–59.4]	27.7 [17.3–40.2]
		Specificity	93 [89.7–95.4]	89.8 [86–92.8]	95 [92.5–96.8]	97.2 [95.1–98.5]	95.2 [92.9–96.9]	95.3 [92.5–97.3]	98.3 [96.7–99.3]	97.7 [95.9–98.9]
		PPV	51 [38–63.8]	62.5 [53.2–71]	48.8 [35.7–62.1]	36.9 [19.2–59.1]	17.2 [9.1–30.2]	82.2 [73.5–88.5]	57.8 [37.5–75.8]	64.2 [46.5–78.8]
		NPV	69.3 [67.7–70.7]	72.3 [70–74.4]	90.1 [88.5–91.5]	84 [83.1–84.9]	99 [90.1–99.4]	77.7 [75.3–80]	96.5 [95.4–97.4]	90.2 [88.8–91.5]
		AUC	0.540 [0.50–0.58]	0.615 [0.57–0.66]	0.632 [0.59–0.67]	0.527 [0.48–0.57]	0.726 [0.68–0.76]	0.698 [0.66–0.74]	0.688 [0.65–0.73]	0.627 [0.58–0.67]
	Reader 2	Sensitivity	25.3 [18.9–32.6]	40 [32.7–47.7]	37.3 [25.8–50]	8.2 [3.4–16.2]	40 [12.2–73.8]	53.9 [46–61.6]	35.7 [18.6–55.9]	41.5 [29.4–54.4]
		Specificity	89.7 [86–92.8]	86.5 [82.3–89.9]	90.9 [87.8–93.4]	98.3 [96.6–99.3]	99.6 [98.6–100]	92.1 [88.7–94.7]	99.8 [98.8–100]	96.8 [94.7–98.3]
		PPV	54.5 [44.3–64.3]	60.3 [52.3–67.8]	38.4 [28.9–48.9]	50.1 [26.5–73.6]	66.6 [29.2–90.6]	76.9 [69.3–83.1]	90.9 [56.9–98.7]	65.8 [51.6–77.7]
		NPV	71.2 [69.2–73.1]	73.7 [71.1–76.1]	90.5 [88.8–92]	84.2 [83.3–85]	98.8 [98–99.3]	80.3 [77.5–82.8]	96.4 [95.3–97.2]	91.9 [90.2–93.3]
		AUC	0.575 [0.53–0.62]	0.632 [0.59–0.67]	0.641 [0.60–0.68]	0.533 [0.49–0.58]	0.698 [0.66–0.74]	0.730 [0.69–0.77]	0.678 [0.64–0.72]	0.692 [0.65–0.73]
	Reader 3	Sensitivity	50 [42.2–57.9]	38.9 [31.6–46.5]	20.9 [11.9–32.6]	14.1 [7.5–23.4]	60 [26.2–87.9]	52.7 [44.8–60.5]	46.4 [27.5–66.1]	47.7 [35.2–60.5]
		Specificity	78 [71.1–80.4]	91.6 [88–94.3]	95.7 [93.3–97.4]	96.2 [93.9–97.8]	95.8 [93.6–97.4]	88.2 [84.3–91.5]	99.4 [98.2–99.9]	93.9 [91.2–95.9]
		PPV	50.3 [44.2–56.3]	70.4 [61.4–78]	42.4 [27.9–58.3]	42.9 [27–60.5]	22.2 [12.9–35.5]	68.7 [61.4–75.2]	81.2 [56.6–93.5]	53.4 [42.3–64.2]
		NPV	75.8 [72.6–78.6]	74.4 [72–76.7]	88.8 [87.5–90]	84.7 [83.6–85.8]	99.2 [98.2–99.6]	79.2 [76.3–81.8]	97 [95.8–97.8]	92.4 [90.6–93.9]
		AUC	0.630 [0.59–0.67]	0.652 [0.61–0.70]	0.583 [0.54–0.63]	0.552 [0.51–0.60]	0.779 [0.74–0.81]	0.705 [0.66–0.74]	0.729 [0.69–0.77]	0.70 [0.67–0.75]
Setting 3	Reader 1	Sensitivity	14.5 [9.5–20.7]	30.9 [24.1–38.3]	23.9 [14.3–35.9]	10.6 [5–19.2]	50 [18.7–81.3]	38.3 [30.9–46.2]	35.7 [18.6–55.9]	21.5 [12.3–33.5]
		Specificity	94.7 [91.8–96.8]	89.5 [85.6–92.6]	95.9 [93.6–97.6]	97.6 [95.7–98.9]	96 [93.9–97.5]	98.5 [96.6–99.5]	100 [99.2–100]	97.1 [95–98.4]
		PPV	57.1 [42.6–70.4]	60.1 [50.7–68.9]	47 [32.3–62.3]	47.4 [27.4–68.3]	20 [10.5–34.7]	92.7 [84–96.9]	100 [N.A.]	51.8 [34.6–68.6]
		NPV	69.5 [68.1–70.9]	71.5 [69.3–73.6]	89.2 [87.9–90.5]	84.4 [83.4–85.4]	99 [90.1–99.4]	76.5 [74.3–78.6]	96.4 [95.3–97.2]	89.4 [88.1–90.6]
		AUC	0.546 [0.50–0.59]	0.602 [0.56–0.64]	0.599 [0.56–0.64]	0.541 [0.50–0.59]	0.730 [0.69–0.77]	0.684 [0.64–0.73]	0.679 [0.64–0.72]	0.593 [0.55–0.64]
	Reader 2	Sensitivity	15.1 [10–21.4]	37.7 [30.5–45.3]	25.4 [15.5–37.5]	8.2 [3.4–16.2]	30 [6.7–66.3]	46.7 [39–54.6]	25 [10.7–44.9]	35.4 [23.9–48.2]
		Specificity	94.4 [91.4–96.6]	86.8 [82.6–90.2]	94.1 [91.5–96.1]	97.9 [96–99]	98.8 [97.4–99.6]	92.9 [89.7–95.4]	99.6 [98.5–100]	97.5 [95.6–98.8]
		PPV	56.8 [42.7–69.8]	59.5 [51.2–67.2]	39.5 [27.3–53.2]	43.8 [23–67.1]	33.3 [12.7–63.2]	76.4 [68.1–83.1]	77.7 [43.1–94.1]	67.6 [51.7–80.3]
		NPV	69.6 [68.1–71]	73 [70.5–75.4]	89.2 [87.8–90.5]	84.1 [83.2–84.9]	98.6 [97.9–99.1]	78.1 [75.5–80.4]	95.8 [94.9–96.6]	91.1 [89.6–92.5]
		AUC	0.547 [0.50–0.59]	0.622 [0.58–0.67]	0.597 [0.55–0.64]	0.531 [0.49–0.58]	0.644 [0.60–0.69]	0.698 [0.66–0.74]	0.623 [0.58–0.67]	0.664 [0.62–0.71]
	Reader 3	Sensitivity	50 [42.2–57.9]	41.1 [33.8–48.8]	13.4 [6.3–24]	14.1 [7.5–23.4]	60 [26.2–87.9]	53.3 [45.4–61]	53.6 [33.9–72.5]	47.7 [35.2–60.5]
		Specificity	73.3 [68.3–77.9]	91.3 [87.7–94.1]	97.1 [95–98.4]	96.9 [94.8–98.4]	95.9 [93.6–97.4]	88.2 [84.3–91.5]	99.8 [98.8–100]	92.5 [89.7–94.8]
		PPV	47.7 [41.9–53.5]	70.8 [62.2–78.2]	40.9 [23.5–60.9]	48.1 [30.4–66.2]	22.2 [12.9–35.5]	69 [61.6–75.4]	93.7 [67.2–99.1]	48.4 [38.2–58.7]
		NPV	75.1 [71.9–78.1]	75.1 [72.6–77.4]	88.1 [87–89]	84.8 [83.7–85.9]	99.2 [98.2–99.6]	79.4 [76.5–82]	97.4 [96.2–98.2]	92.3 [90.5–93.8]
		AUC	0.617 [0.57–0.66]	0.662 [0.62–0.70]	0.552 [0.51–0.60]	0.555 [0.51–0.60]	0.779 [0.74–0.81]	0.708 [0.67–0.75]	0.767 [0.73–0.80]	0.701 [0.66–0.74]
Setting 4	Reader 1	Sensitivity	17.5 [12–24.1]	40 [32.7–47.7]	23.4 [15.5–37.5]	11.8 [5.8–20.6]	50 [18.7–81.3]	46.7 [39–54.6]	46.4 [27.5–66.1]	29.2 [18.6–41.8]
		Specificity	93.8 [90.7–96.2]	87.1 [83–90.5]	95.7 [93.3–97.4]	97.4 [95.4–98.7]	95.4 [93.1–97]	97.4 [95–98.8]	100 [99.2–100]	95.5 [93.1–97.2]
		PPV	58 [44.8–70.1]	61.4 [53.3–68.9]	47.2 [32.9–62]	47.7 [28.6–67.5]	17.8 [9.4–31.2]	89.6 [81.7–94.4]	100 [N.A.]	48.7 [34.9–62.7]
		NPV	70.1 [68.5–71.6]	73.8 [71.3–76.2]	89.4 [88–90.7]	84.5 [83.5–85.5]	99 [90.1–99.4]	78.8 [76.4–81.1]	97 [95.9–97.8]	90.2 [88.7–91.5]
		AUC	0.557 [0.51–0.60]	0.635 [0.59–0.68]	0.605 [0.56–0.65]	0.546 [0.50–0.59]	0.727 [0.69–0.77]	0.720 [0.68–0.76]	0.732 [0.69–0.78]	0.624 [0.58–0.67]
	Reader 2	Sensitivity	15.7 [10.5–22.1]	39.4 [32.1–47.1]	23.9 [14.3–35.9]	8.2 [3.4–16.2]	40 [12.2–73.8]	47.3 [39.5–55.2]	25 [10.7–44.9]	36.9 [25.3–49.8]
		Specificity	94.7 [91.8–96.8]	85.8 [81.6–89.4]	93.9 [91.2–95.9]	97.6 [95.7–98.9]	98.8 [97.4–99.6]	92.9 [89.7–95.4]	99.6 [98.5–100]	97.1 [95–98.4]
		PPV	59.1 [44.9–71.9]	58.9 [51–66.5]	37.2 [25.2–51]	41.2 [21.6–64.2]	40 [18.2–66.7]	76.7 [68.4–83.3]	77.7 [43.1–94.1]	64.8 [49.7–77.5]
		NPV	69.8 [68.3–71.3]	73.3 [70.8–75.8]	89 [87.6–90.3]	84.1 [83.2–84.9]	98.8 [98–99.3]	78.3 [75.7–80.6]	95.8 [94.9–96.6]	91.3 [89.7–92.7]
		AUC	0.552 [0.51–0.60]	0.626 [0.58–0.67]	0.589 [0.54–0.63]	0.529 [0.49–0.57]	0.694 [0.65–0.73]	0.701 [0.66–0.74]	0.623 [0.58–0.67]	0.670 [0.63–0.71]
	Reader 3	Sensitivity	50 [42.2–57.9]	47.4 [39.9–55.1]	22.4 [13.1–34.2]	17.7 [10.2–27.4]	60 [26.2–87.9]	53.3 [45.4–61]	53.6 [33.9–72.5]	47.7 [35.2–60.5]
		Specificity	73 [68–77.7]	90.4 [88.7–93.3]	94.1 [91.5–96.1]	96 [93.6–97.6]	95.8 [93.6–97.4]	88.2 [84.3–91.5]	99.8 [98.8–100]	92.5 [89.7–94.8]
		PPV	47.4 [41.7–53.2]	71.7 [63.8–78.5]	36.6 [24.4–50.8]	46.9 [31.5–63]	22.2 [12.9–35.5]	69 [61.6–75.4]	93.7 [67.2–99.1]	48.4 [38.2–58.7]
		NPV	75 [71.8–78]	76.9 [74.3–79.4]	88.9 [87.5–90.1]	85.2 [83.9–86.5]	99.2 [98.2–99.6]	79.4 [76.5–82]	97.4 [96.2–98.2]	92.3 [90.5–93.8]
		AUC	0.615 [0.57–0.66]	0.689 [0.65–0.73]	0.582 [0.54–0.63]	0.568 [0.52–0.61]	0.779 [0.74–0.81]	0.708 [0.67–0.75]	0.767 [0.73–0.80]	0.701 [0.66–0.74]

### Interobserver agreement

*K*_*w*_ values for any finding ranged 0.23–0.63, 0.13–0.73, 0.21–0.66, 0.14–0.75 for settings 1, 2, 3 and 4, respectively. Regardless of size, interobserver agreement at the detection of pneumothorax between the residents and the senior radiologist showed a slight improvement in both settings 3 and 4 (Table 3). *K*_*w*_ values were generally higher for large pneumothorax—sized ≥ 3 cm [29]—with only two exceptions of greater values observed for small pneumothoraces (sized < 3 cm). Details are reported in Table 4.

**Table 3:** Interobserver agreement between the three Readers (Reader 1: experienced radiologist; Readers 2 and 3: radiology residents)

	Reader 1 vs. Reader 2				Reader 1 vs. Reader 3				Reader 2 vs. Reader 3			
	Setting 1 Kappa value [95%CI]	Setting 2 Kappa value [95%CI]	Setting 3 Kappa value [95%CI]	Setting 4 Kappa value [95%CI]	Setting 1 Kappa value [95%CI]	Setting 2 Kappa value [95%CI]	Setting 3 Kappa value [95%CI]	Setting 4 Kappa value [95%CI]	Setting 1 Kappa value [95%CI]	Setting 2 Kappa value [95%CI]	Setting 3 Kappa value [95%CI]	Setting 4 Kappa value [95%CI]
Atelectasis	0.29 [0.17–0.40]	0.34 [0.20–0.48]	0.28 [0.16–0.40]	0.28 [0.14–0.40]	0.23 [0.15–0.31]	0.13 [0.06–0.2]	0.21 [0.13–0.29]	0.14 [0.07–0.22]	0.33 [0.24–0.41]	0.17 [0.1–0.24]	0.31 [0.23–0.4]	0.15 [0.08–0.22]
Consolidation	0.36 [0.27–0.46]	0.38 [0.28–0.48]	0.38 [0.28–0.47]	0.42 [0.33–0.51]	0.34 [0.25–0.44]	0.35 [0.25–0.45]	0.38 [0.28–0.47]	0.34 [0.25–0.44]	0.44 [0.34–0.54]	0.41 [0.31–0.5]	0.42 [0.33–0.52]	0.42 [0.33–0.52]
Interstitial abnormalities	0.38 [0.25–0.5]	0.34 [0.2–0.49]	0.36 [0.24–0.49]	0.33 [0.18–0.47]	0.35 [0.2–0.49]	0.32 [0.16–0.49]	0.35 [0.2–0.49]	0.37 [0.22–0.51]	0.32 [0.19–0.45]	0.43 [0.27–0.58]	0.33 [0.2–0.46]	0.43 [0.29–0.57]
Nodule	0.47 [0.24–0.7]	0.32 [0.11–0.53]	0.47 [0.25–0.68]	0.29 [0.09–0.49]	0.39 [0.20–0.58]	0.29 [0.1–0.47]	0.44 [0.26–0.62]	0.34 [0.17–0.52]	0.37 [0.17–0.57]	0.37 [0.17–0.56]	0.41 [0.22–0.6]	0.30 [0.12–0.47]
Mass	0.25 [0.07–0.44]	0.34 [0.13–0.54]	0.21 [0.03–0.39]	0.30 [0.1–0.5]	0.40 [0.23–0. 57]	0.43 [0.26–0.6]	0.43 [0.26–0.6]	0.40 [0.23–0.57]	0.30 [0.09–0.5]	0.32 [0.12–0.51]	0.23 [0.04–0.42]	0.36 [0.16–0.56]
Pleural effusion	0.62 [0.54–0.71]	0.56 [0.46–0.66]	0.61 [0.52–0.69]	0.60 [0.51–0.69]	0.55 [0.46–0.64]	0.50 [0.41–0.59]	0.50 [0.41–0.59]	0.51 [0.42–0.6]	0.52 [0.43–0.61]	0.47 [0.38–0.56]	0.53 [0.45–0.62]	0.50 [0.41–0.59]
Pneumothorax	0.52 [0.3–0.74]	0.73 [0.51–0.96]	0.59 [0.38–0.8]	0.63 [0.39–0.87]	0.50 [0.29–0.7]	0.68 [0.48–0.89]	0.56 [0.36–0.76]	0.75 [0.57–0.93]	0.63 [0.41–0.85]	0.55 [0.31–0.79]	0.66 [0.45–0.87]	0.55 [0.31–0.79]
Rib fractures	0.54 [0.39–0.69]	0.42 [0.26–0.59]	0.55 [0.41–0.7]	0.49 [0.34–0.63]	0.38 [0.24–0.53]	0.35 [0.22–0.48]	0.40 [0.26–0.53]	0.43 [0.31–0.56]	0.51 [0.38–0.64]	0.37 [0.25–0.5]	0.54 [0.42–0.67]	0.40 [0.27–0.53]

**Table 4:** Interobserver agreement between the three Readers for small and large pneumothoraces

	Reader 1 vs. Reader 2				Reader 1 vs. Reader 3				Reader 2 vs. Reader 3			
	Setting 1 Kappa value [95%CI]	Setting 2 Kappa value [95%CI]	Setting 3 Kappa value [95%CI]	Setting 4 Kappa value [95%CI]	Setting 1 Kappa value [95%CI]	Setting 2 Kappa value [95%CI]	Setting 3 Kappa value [95%CI]	Setting 4 Kappa value [95%CI]	Setting 1 Kappa value [95%CI]	Setting 2 Kappa value [95%CI]	Setting 3 Kappa value [95%CI]	Setting 4 Kappa value [95%CI]
Pneumothorax < 3 cm	0.27 [0.18–0.72]	0.77 [0.34–1]	0.67 [0.25–1]	0.39 [0.02–0.8]	0.23 [-0.2–0.7]	0.39 [0.03–0.74]	0.61 [0.24–0.97]	0. 25 [0.12–0.91]	0.56 [0.16–0.96]	0.26 [-0.1–0.58]	0.61 [0.24–0.97]	0.26 [-0.1–0.58]
Pneumothorax ≥ 3 cm	0.70 [0.17–1]	0.70 [0.17–1]	0.70 [0.17–1]	0.70 [0.17–1]	0.42 [-0,2–1]	0.42 [-0,2–1]	0.42 [-0,2–1]	0.42 [-0,2–1]	0.70 [0.17–1]	0.70 [0.17–1]	0.70 [0.17–1]	0.70 [0.17–1]

## Discussion

We observed that grey-scale inversion display mode did not significantly improve diagnostic performance or interobserver agreement compared with standard viewing mode. Combinations of standard and inverted modes could help in reducing the interobserver variability across different levels of expertise.

The visualization of CXR is usually performed by “white bones” mode on video-terminal; however, the perception of CXR images is also (variably) preferred with “black bones” mode. The latter represents a subjective adaptation of the standard setting, based on the individual feeling that the detection of abnormal findings is eased by the inverted images. We undertook this study for systematic evaluation of such perception and showed that there is no actual diagnostic difference. Our results partially confirm previous observation from Park et al. who investigated sensitivity and accuracy of the grey-scale inversion technique, limited to the detection of rib fractures. Park reported that the combination of the two reading modalities could improve chest radiography sensitivity and accuracy among residents and medical students, namely among readers with limited experience [27]. In our study, the combined use of the two approaches increased CXR sensitivity at the detection of consolidation for one resident, when using reading setting 4 (i.e. first inverted, followed by standard), pneumothorax and rib fractures in setting 3 (i.e. first standard, followed by inverted) and 4. However, the improvement did not reach statistical significance for accuracy performance.

Interobserver agreement at the detection of pneumothorax between the residents and the senior radiologist showed a moderate improvement in both sessions and, as expected, was generally higher for large pneumothoraces in all settings and among all readers. Since the required reading time for both sessions was relatively short (not greater than 84 s), the combined use of the two display modes might be worth exploiting when pneumothorax is suspected. Having said that, pneumothorax was scarcely represented among the enrolled patients (5.5%, 28 cases).

The combined reading approach improved the PPV at the detection of pleural effusion by the senior radiologist, but showed a general drop in diagnostic performance as compared to the standard approach for the same reader. The unfamiliarity with the “black bones” images might have affected their interpretation by the senior radiologist. As pointed out by McMahon et al., when a new type of image results in lesser accuracy, the unfamiliarity with the new approach must be taken into account prior to blaming intrinsic properties of the new modality [11]. This “unfamiliarity effect” tends to have a minor impact on younger author radiologists, who are inevitably less affected by a long-lasting habit.

Thompson et al. reported that two display modes can improve nodule detection [26]. These authors hypothesized that the advantage of using two display modes might lie in the fast-flicking between the two images, namely standard and inverted, which would draw attention to suspicious areas, (e.g. lung periphery). This fast-flicking technique was not employed by our readers, for whom the detection was already slightly improved, suggesting that it might only partly explain the advantages of such a combination. Even if limited in number, the majority of studies that have applied the grey-scale inversion display mode to chest radiography have attempted to demonstrate its additional value in detecting lung nodules, either real or simulated, with opposite results [17, 22–26]. Nodules were fairly represented in our sample (16.8%, 85 cases), and significant differences were not observed in accuracy or interobserver agreement with the combination of the two techniques. Their depiction rate was generally low among the three readers, ranging 7.1% to 17.7%. One of the reasons of such low percentages can be found in their relatively small size (nodule median diameter of 7 mm, 95%CI, 6 to 8 mm), which has likely contributed to reduce their detectability by CXR. Previous studies reported better performance in nodule detection, notably with relatively larger solid nodules [17]. As opposite to previous analyses, a nodule size range was not set at the time of patient selection (22), since the general intent of this investigation was to reproduce a real clinical setting, without focusing on a pre-defined finding.

To our knowledge, this is the first study testing eight different abnormal findings at the same time and within such a large population. Indeed, the majority of studies that have investigated the application of grey-scale inversion display mode into chest radiography only tested one selected finding at time, enrolling no more than 300 subjects. Furthermore, we included bedside CXRs, with the aim of reproducing a real clinical setting, where a good proportion of patients is unable to stand (e.g. trauma patients or severely ill ones). Of note, the effect of reading setting was comparable for both standing and supine CXR imaging.

Our study, however, has several limitations. First, the retrospective design is prone to confounding factors, such as selection of patients. Second, CXRs were obtained with different technical equipment and parameters, which can ultimately affect the detectability of findings, nonetheless representing the actual routine of this imaging modality. Third, some of the findings included in the analysis were barely represented within the sample, such as mass (1.97%, 10 cases). Finally, the presence of only one senior radiologist limited the possibility of investigating the impact of different levels of expertise.

In conclusion, we observed no significant advantages in the use of grey-scale inversion technique in expert radiologist. The combination of grey-scale inversion display modes with standard mode could reduce the interobserver variability in readers with limited expertise.

## Supplementary Information

Below is the link to the electronic supplementary material.

Supplementary file1 (DOCX 14 kb)

## Data Availability

All data generated or analysed during this study are included in this published article and its supplementary information file.

## Abbreviations

AP: Anteroposterior
AUC: Area under the curve
CT: Computed tomography
CXR: Chest X-ray
LL: Left-lateral
NPV: Negative predictive value
PA: Posteroanterior
PACS: Picture archiving and communication system
PPV: Postive predictive value
