# Supplementary material for: The diagnostic value of grey-scale inversion technique in chest radiography
Source: Radiol Med. 2022 Jan 18;127(3):294–304. doi: 10.1007/s11547-022-01453-0 (PMC8960630; doi:10.1007/s11547-022-01453-0)
Supplement: Supplementary file 1 — Supplementary file1 (DOCX 14 kb) [file 11547_2022_1453_MOESM1_ESM.docx]

## **Supplementary material**

### CT imaging technique – Standard of reference

Enhanced and unenhanced chest CTs were performed with four different CT scanners (two 128-slice scanner: SOMATOM Definition Edge and SOMATOM Definition Flash, Siemens Healthineers, Erlangen, Germany; a 64-slice scanner: SOMATOM Sensation, Siemens Healthineers, Erlangen, Germany and a 6-slice scanner: SOMATOM Emotion, Siemens Healthineers, Erlangen, Germany). Images were acquired with the patient in the supine position during end-inspiration breath-hold. The acquisition parameters were 100-140 kVp and 80 reference mAs. Enhanced CTs images were obtained after the intravenous injection of iodinated contrast agent (50–120 mL apportioned to the body weight and clinical indication, injection rate 2.5–4 mL/s) through the ante-cubital vein followed by a saline chaser (20–40 mL), using a double syringe injector. Reconstruction parameters for lung images were as following: slice thickness ≤ 2.5 mm, lung window (width, 1600 Hounsfield Units, HU; level, −600 HU), whereas for mediastinal images: slice thickness 2.0 mm, mediastinal window (width, 400 HU; level, 30 HU).

### CT acquisition data

One hundred and twenty out of 507 (23.7%, 95%CI 20.18% to 27.56%) CTs were unenhanced; 329/507 (64.9%, 95%CI 60.64% to 68.92%) both unenhanced and enhanced and 58/507 (11.4%, 95%CI 8.95% to 14.51%) only enhanced. One hundred and fifty nine out of 507 (31.4%, 95% CI 27.47% to 35.53%) were reconstructed with a minimum slice thickness of 1mm; 283/507 (55.8%, 95%CI 51.47% to 60.08%) of 1.5mm; 63 (12.4%, 95%CI 9.84% to 15.59%) of 2mm; 1 (0.2%, 95%CI 0.04% to 1.1%) of 1.25mm and 1 (0.2%, 95%CI 0.04% to 1.1%) of 2.5mm.
